# Recent advances in antigen targeting to antigen-presenting cells in veterinary medicine

**DOI:** 10.3389/fimmu.2023.1080238

**Published:** 2023-03-10

**Authors:** Edgar Alonso Melgoza-González, Lorena Bustamante-Córdova, Jesús Hernández

**Affiliations:** Laboratorio de Inmunología, Centro de Investigación en Alimentación y Desarrollo A. C., Hermosillo, Mexico

**Keywords:** antigen target, antigen presenting cell, receptors, veterinary, vaccines

## Abstract

Advances in antigen targeting in veterinary medicine have gained traction over the years as an alternative approach for diseases that remain a challenge for traditional vaccines. In addition to the nature of the immunogen, antigen-targeting success relies heavily on the chosen receptor for its direct influence on the elicited response that will ensue after antigen uptake. Different approaches using antibodies, natural or synthetic ligands, fused proteins, and DNA vaccines have been explored in various veterinary species, with pigs, cattle, sheep, and poultry as the most frequent models. Antigen-presenting cells can be targeted using a generic approach, such as broadly expressed receptors such as MHC-II, CD80/86, CD40, CD83, etc., or focused on specific cell populations such as dendritic cells or macrophages (Langerin, DC-SIGN, XCR1, DC peptides, sialoadhesin, mannose receptors, etc.) with contrasting results. Interestingly, DC peptides show high specificity to DCs, boosting activation, stimulating cellular and humoral responses, and a higher rate of clinical protection. Likewise, MHC-II targeting shows consistent results in enhancing both immune responses; an example of this strategy of targeting is the approved vaccine against the bovine viral diarrhea virus in South America. This significant milestone opens the door to continuing efforts toward antigen-targeting vaccines to benefit animal health. This review discusses the recent advances in antigen targeting to antigen-presenting cells in veterinary medicine, with a special interest in pigs, sheep, cattle, poultry, and dogs.

## 1 Introduction

Antigen-presenting cells (APCs), such as macrophages, dendritic cells (DCs), and B lymphocytes, are a fundamental part of the innate immune system and play essential roles in initiating and regulating the adaptive response (1, 2). Its main function is recognizing, capturing, and processing antigens and presenting immunogenic peptides to naïve T lymphocytes to initiate the adaptive cellular immune response (3–6). Antigen recognition and internalization are mediated by receptors on the surface of APCs. Through this mechanism of antigen capture, antigen-targeting strategies have been developed to enhance vaccine efficiency and have been widely explored for the last two decades as prophylactic and therapeutic tools for infectious diseases, autoimmunity and cancer (7–15).

**Figure 1:**
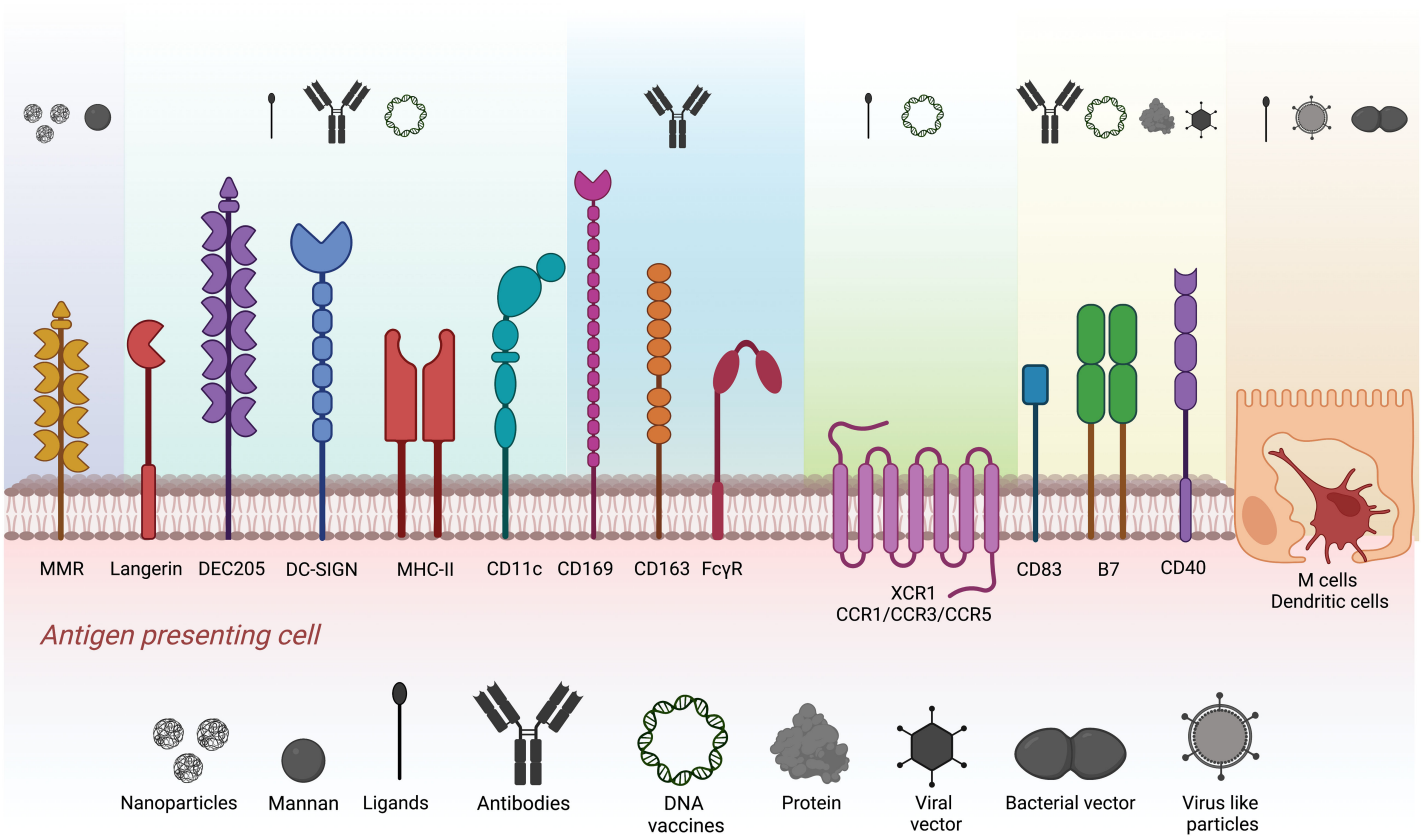
Strategies explored for antigen targeting to different APC populations. Target surface receptors on APCs and M cells evaluated in antigen targeting. Different colors represent the clusters of carriers such as nanoparticles, mannan, ligands, antibodies, DNA vaccines, proteins, virus-like particles, and viral and bacterial vectors used to target specific surface receptors.

The success of antigen-targeting strategies heavily relies on selecting the target receptor, the antigen being delivered, and the antigen carrier. Along with choosing a specific target receptor, the combination of the APC target and adjuvant utilized contributes to the polarization of the CD4^+^ T lymphocyte response toward the Th1, Th2, Th17, or Treg profile (16–22). These characteristics play a key role in the immune response for future pathogen clearance. Several types of surface receptors are the focus of antigen-targeting research. Pattern-recognizing receptors, chemokine receptors, costimulatory molecules, and cell adhesion receptors are the most common. Interestingly, only a few receptors are known to be highly expressed or almost exclusive to a cell type, such as XCR1, Langerin, DEC205, and DC-SIGN for DCs or CD169, MMR, and CD163 for macrophages. Other molecules, such as MHC-II, CD80/86, CD40, CD83, and CD11c, are widely expressed by a variety of APCs. Among the most popular strategies to shape immune responses is using natural ligands such as glycans to target C-type lectin receptors or proteins (recombinant ligands or antibodies) that recognize surface receptors on APCs (**Figure 1**). DNA vaccines codifying recombinant proteins fused to the antigen of interest have also been evaluated (23–29). Likewise, the route of administration greatly impacts the development of systemic or mucosal responses, where intradermal, subcutaneous, intramuscular, and oral are the most common immunization routes (30–33).

The diversity of target receptors, antigens, carriers, adjuvants, and administration routes allows for the customization of targeting vaccine strategies to stimulate different aspects of the immune response and will directly impact the level of protection in the different animal species (**Supplementary Table 1**). However, to date, most evidence supporting antigen targeting has been produced using mice and guinea pigs as transitory models for humans. For this reason, the present review aimed to explore the different approaches and strategies reported encompassing the evaluation of antigen targeting as an immunoprophylactic tool in species of veterinary importance (**Table 1**) and not just animal models used as surrogates in human medical research.

**Table 1:** Reports of antigen targeting evaluations in common veterinary species.

Target	Target species						
	*Sheep*	*Swine*	*Cattle*	*Poultry*	*Dogs*	*Ferret*	Rodents*
B7	(34–37)	(38–40)	(41)	–	–	–	(40, 42, 43)
CCR1/3/5	–	(44)	–	(45)	–	–	–
CD11c	(46)	(47, 48)	–	(49)	–	–	–
CD163	–	(50)	–	–	–	–	–
CD40	(51, 52)	–	(53)	(54–56)	(57)	–	–
CD83	–	–	–	(58, 59)	–	–	–
DCs	–	(60–65)	(66)	(67–71)	–	–	(60, 65, 66, 68, 72)
DC-SIGN	–	(73, 74)	–	–	–	–	–
DEC205	(46, 75)	(73, 76, 77)	(25)	(49, 78–80)	–	–	(75)
FcγR	–	(81)	–	–	–	–	–
Langerin	–	(73, 82, 83)	–	–	–	–	–
M cells	–	–	–	–	–	–	(84)
MMR	–	(85–87)	–	–	–	–	–
MHC-II	–	(24, 88, 89)	(90–94)	–	(95)	(24)	(24, 91–95)
Sialoadhesin	–	(50, 96, 97)	–	–	–	–	–
XCR1	–	(48, 98, 99)	(100)	–	–	–	–

*Included mice, rabbits, and guinea pigs. We included rodents and ferrets since they were used to evaluate antigen-targeting vaccines for veterinary medicine purposes. The extended information is listed in detail in **Supplementary Table 1**. Symbol "-" represents the absence of antigen-targeting reports in those animal species.

## 2 Targeting using the C-type lectin receptor family

### 2.1 CLRs type I

#### 2.1.1 DEC205

This endocytic receptor is predominantly expressed in dendritic cells, although it has also been reported in various cell types, such as macrophages, T lymphocytes, and B lymphocytes, with differential expression between species (101–104). In addition, it has been characterized in species such as mice, humans, sheep, cattle, and pigs (105–108). DEC205 can promote cross-presentation (the ability to capture, process, and present extracellular antigens with MHC-I to CD8^+^ T cells) and is capable of being recycled, although the coupling of a ligand or antibody does not guarantee the activation or maturation of DCs (105, 109, 110).

Due to its impact on the poultry industry, avian influenza virus (AIV) antigens were targeted to DEC205 to promote an effective immune response in chickens. The targeting strategy consisted of subcutaneous immunization using an anti-DEC205 antibody to target AIV hemagglutinin protein (HA) to DEC205^+^ cells. The results showed a significant improvement in the humoral response, evidenced by early production and higher levels of total and neutralizing antibodies (NAbs) in sera (49, 78). No effect on proinflammatory cytokines such as IFN-γ, IL-6, and IL-1β was observed. Following a similar strategy, the HN antigen of the Newcastle disease virus (NDV) was targeted to DEC205^+^ cells, resulting in enhanced production of total and NAbs compared with the nontargeted group (79). On the other hand, in a tumoral model induced by Rous sarcoma virus (RSV), subcutaneous targeting to DEC205 skewed the cytokine profile toward the Th1 response, as evidenced by an increase in IL-12, IL-2, and IFN-γ triggering the cellular immune response against the tumor (80).

In sheep, the intradermal injection of a DNA vaccine encoding an anti-DEC205 scFv fused with the Gn and Gc antigens of Rift Valley fever (RVFV) along with granulocyte-macrophage colony-stimulating factor (GM-CSF) promoted a higher frequency of IFN-γ^+^ T lymphocytes and lower antibody titers compared to the nontargeted antigens (46). Contrary to the results in sheep, the murine counterpart for this study showed no improvement in the humoral response against RVFV, nor had any effect on the cellular response (75). Therefore, even though the strategy was the same, antigen targeting can differ between species, resulting in unpredictable immune responses. In cattle, the intradermal application of a DNA vaccine encoding an anti-DEC205, coupled with the CD40L activation domain and B and T-cell epitopes of *Anaplasma marginale* Merozoite Surface Protein-1 (MSP1), showed promising results. The proliferative response of CD4^+^ T cells, IFN-γ production, and total IgG titers were significantly increased after a single application and increased after a second exposure (25).

In swine, there are limited and contrasting reports about the efficacy of DEC205^+^ DC targeting. Evaluating the targeting of GP3, GP4, GP5, and M from PRRSV toward DCs through the intramuscular route promotes the response of CD4^+^CD8^+^ T lymphocytes positive for IFN-γ and IL-4, although it failed to stimulate the humoral response. These results were not significantly different from the nontargeting antigen group. As the authors mentioned, the immunization route could not be appropriate to target DCs because their presence in this tissue might be scarce (73). Afterward, Bustamante-Córdova et al. (76) evaluated the effect of targeting immunogenic peptides from PRRSV to intradermal DEC205^+^ DCs. They found a higher antigen-specific IgG response compared to the control group but with no differences in T lymphocytes IFN-γ^+^ (76). These results suggest that the route of administration can affect the induction of humoral or cellular immune responses. As a follow-up study, Melgoza-González et al. (77) evaluated antigen targeting using porcine circovirus 2 (PCV2) capsid protein (Cap). The cellular response of IFN-γ^+^ CD4^+^CD8^+^ lymphocytes was enhanced compared to the control group, with a discrete effect on the humoral response (77). These results also suggest that antigen targeting using DEC-205 can stimulate a differential response according to the antigen used, highlighting the importance of the antigen in this kind of immunization system.

#### 2.1.2 Macrophage mannose receptor

MMR is a surface endocytic and phagocytic receptor expressed on macrophages and some myeloid DC subsets (111). This receptor possesses multiple carbohydrate recognition domains that can bind to mannan and fucose from exogenous antigens, playing a crucial role in the innate immune response (112).

To enhance antigen uptake by APCs, antigens targeting the mannose receptor have been explored in various studies. Mice immunized intradermally with mannosylated PCV2 nanoparticles presented higher levels of IgG, IL-4, and IL-2 than the nontargeted group, even in the absence of other adjuvants. Additionally, the study showed that the mannosylated protein presented a slow release when exposed to low pH *in vitro* , simulating lysosome conditions and furthering the potential of using mannosylation as a controlled-release tool for drugs (85).

In addition, the mannosylation of gelatin nanoparticles (MnGNPs) encapsulating inactivated PRRSV significantly improved antigen uptake compared with nonmannosylated gelatin particles by up to 15 times. Additionally, MnGNPs were capable of boosting the expression of SWC-3a, CD80, CD1, SLA-I, and SLA-II markers in monocyte-derived DCs (moDCs). The production levels of IL-1β, IL-6, IL-10, and IL-12 were significantly enhanced by MnGNPs, as was the specific cytotoxic T-cell activity. Further exploration of this strategy *in vivo* studies could position mannose receptor targeting as a prime candidate to aid vaccination efforts against otherwise difficult pathogens (86).

In addition to uptake enhancement, mannose receptor targeting through mannosylation of antigens has been proposed to be able to circumvent the detrimental effect of maternal-derived antibodies (MDA) in the vaccination of young animals (87). For this, mannosylated chitosan-based nanoparticles encapsulating swine influenza virus (SIV) antigens were administered intranasally in piglets following a prime-boost regimen. The strategy successfully enhanced heterologous and homologous IgA responses in the nasal mucosa and the respiratory system. Moreover, the mannosylated vaccine induced higher antigen-specific cell proliferation and IFN-γ expression than the commercial vaccine. In addition, significantly lower viral shedding, lower viral load in bronchoalveolar fluid and lung lysate along with fewer lung lesions were observed (87). In conclusion, the mannosylation of SIV antigens effectively elicited a robust and protective immune response in piglets despite the presence of MDA, highlighting its potential as a valuable vaccination strategy.

### 2.2 Type II CLRs

#### 2.2.1 DC-SIGN (CD209)

This receptor can bind to mannose and fucose residues and is capable of not only recognizing but also internalizing several pathogens, such as *Mycobacterium tuberculosis, Candida albicans and Leishmania spp.,* among others (113–115). Although DC-SIGN expression is believed to be restricted to DCs, it is also expressed by macrophages (116, 117). Interestingly, as with other receptors from the CLR family, DC-SIGN enables cross-presentation (118, 119). The efficacy of antigen targeting to the porcine DC-SIGN receptor was first evaluated using a chimeric mouse x pig mAb anti-DC-SIGN fused to antigenic peptides from PRRSV using monophosphoryl-lipid A (MPLA) as an adjuvant and administered intradermally in a prime-boost approach. In this instance, a significant increase in IFN-γ-secreting CD4^+^ and CD4^+^CD8^+^ T cells was observed in the targeted group in comparison with the nontargeted group. Unfortunately, there was no detectable effect on the humoral immune response in immunized pigs (74).

In a follow-up study, the PRRSV-antigenized chimeric mAb was injected intramuscularly and in the presence of Poly I:C as an adjuvant, an agonist of TLR3. This resulted in a modest stimulation of IFN-γ-secreting CD4^+^CD8^+^, IL-4^+^ CD4^+^CD8^+^ T, and IL-4^+^CD8^+^ T cells at 42 days postvaccination compared to the negative control injected with PBS, but no difference was found when compared to the nontargeted group injected with antigens only. Again, no effect was found in the humoral immune response (73). Under the conditions evaluated, intramuscular targeting failed to induce an enhanced IL-4^+^ and IFN-γ^+^ T-cell response over the non-targeting group. The use of other routes of administration could improve the effects of targeting using DC-SIGN, such as the intradermal route due to the abundance of DCs present in the dermis (120), where several subpopulations of DC-SIGN^+^ cells have been previously described in swine (121, 122). In this way, targeting DCs could be enhanced with the possibility of a higher effect of delivering antigens to skin DCs.

#### 2.2.2 Langerin

CD207, also known as Langerin, is a receptor expressed in skin-resident APCs, such as epidermal Langerhans cells, and at lower levels in dermal Langerin^+^ DCs and CD8α^+^ DCs in lymph nodes (123–125). This endocytic receptor recognizes mannose, fucose, N-acetyl mannosamine, etc., via its carbohydrate recognition domain, mediating internalization, antigen processing, and cross-presentation (126–129). In mice, targeting Langerin^+^ DCs triggers a Th1 immune response (101). Intradermal targeting of porcine epidemic diarrhea virus (PEDV) antigens to langerin receptors using cholera toxin as an adjuvant resulted in a significant increase in IFN-γ-secreting CD4^+^CD8^+^ T cells 7 days after vaccination. On the other hand, when administered intramuscularly, the humoral immune response was better stimulated, with higher production of IgG and IgA at 35 and 42 days postvaccination, respectively (82). In line with this, a similar strategy was applied to evaluate whether sow vaccination with targeted PEDV antigens could offer protection to piglets through maternal antibody transfer. In this case, a commercial nondisclosed adjuvant was utilized. While the humoral immune response was not greatly stimulated by antigen targeting, there was an increase in IFN-γ secreting T cells (CD4^+^, CD8^+^, and CD4^+^CD8^+^), IL-4^+^CD4^+^ and IL-4^+^CD4^+^CD8^+^ cells at 7 days postvaccination compared to the commercial vaccine. Unfortunately, these results did not translate into protection for the piglets where clinical signs were similar in all challenged groups regardless of vaccine type (83).

Targeting PRRSV antigens to langerin receptors intramuscularly and in the presence of Poly I:C resulted in poor stimulation of the cellular and humoral immune responses with a slight increase in IL-4^+^ CD8^+^ T cells. Targeting the same antigenic peptides to other C-type receptors, such as DEC-205 and DC-SIGN, was able to better stimulate IFN-γ and IL-4 responses. Moreover, the langerin-targeted group presented higher levels of viremia than the challenged control group in this study (73). Overall, langerin targeting failed to induce robust cellular and humoral responses, providing poor results regarding clinical signs and protection.

## 3 Targeting major histocompatibility class II

MHC-II is expressed on the surface of APCs, displaying exogenous antigens for antigen presentation to CD4^+^ T lymphocytes (130, 131). MHC-II can be recycled from the cell surface and tagged for degradation into early endosomes with the possibility of promoting cross-presentation by CD8a^+^ DCs (132–134).

Intradermal targeting of bovine MHC-II using an invariant chain motif coupled to MSP1 antigen along with the molecular adjuvants FLT3L and GM-CSF was evaluated in calves. The strategy resulted in enhanced proliferation of CD4^+^ lymphocytes, a higher frequency of IFN-γ-secreting cells, and higher antibody IgG levels with a fast and robust recall response (90). This approach aimed to target intracellular MHC-II molecules in the endosome-lysosome stage during the antigen processing pathway.

Similarly, the APCH1 single chain fragment variable (scFv) antibody has been considered a molecular adjuvant that recognizes an invariant epitope of MHC class II-DR in several species. In swine, APCH1 joined to immunodominant antigens of the African swine fever virus (ASFV) were codified into a DNA vaccine and applied using an intramuscular-subcutaneous prime-boost strategy. Although targeting SLA-II elicited the proliferation of CD4^+^ T cells, IFN-γ-secreting cells, and humoral responses, the latter lacked neutralizing activity and protective immunity against a lethal viral challenge with heterologous strains (88).

Intramuscular targeting of B and T-cell epitopes (BTTs) from foot and mouth disease virus (FMDV) to MHC-II-DR, without additional adjuvants, increased the frequency of IFN-γ secreting cells but did not stimulate the humoral immune response. Nonetheless, after a viral challenge, half of the pigs were partially protected, while the other half had complete protection against clinical signs of disease (89). Additionally, in swine, MHC-II targeting with HA of SIV was enough to stimulate significantly higher IgG and NAbs, while the nontargeting vaccine failed to elicit a humoral response (24).

MHC-II-targeting of enveloping E2 antigen from bovine viral diarrhea virus (BVDV) intramuscularly in guinea pigs and cattle promoted higher NAb titers. This humoral immune response was sufficient to promote total protection against a viral challenge, preventing the development of clinical signs (91). Moreover, the enhanced levels of NAbs in response to the targeted group were similar in titer and protection efficacy to the inactivated vaccine, even under field conditions. This strategy was approved as the first antigen-targeting vaccine commercially available in Peru and Argentina (92, 93). Later, VP2 of the Bluetongue virus (BTV) was coupled to APCH1 and used for intramuscular vaccination in guinea pigs, cattle, and mice. Four times lower amounts of antigens targeted through APCH1 elicited similar NAbs titers than the free VP2 antigen group in guinea pigs and cattle (94). In rabbits and mice, intramuscular targeting of VP60 from rabbit hemorrhagic disease virus (RHDV) mediated by APCH1 fusion protein provided protection after a viral challenge, allowing postchallenge survival (95). Clearly, targeting MHC-II in APCs, independent of the cell type and antigen delivered, seems to be an efficient strategy for the induction of humoral and cellular immune responses, promoting partial to complete protection after a challenge. It is worth mentioning that antigen targeting to MHC-II allows not only DCs to gain access to the antigen but also stimulates B lymphocytes, thus effectively activating cellular and humoral immune responses. These promising results and an approved vaccine in the market put this strategy at the forefront of antigen-targeting-based immunoprophylactic tools.

## 4 Targeting activation markers

### 4.1 CD40

CD40 is a surface costimulatory receptor from the tumor necrosis factor receptor family. It is expressed in monocytes, macrophages, B-lymphocytes, dendritic cells, and endothelial and epithelial cells (135). The interaction of CD40 and CD40L (expressed on CD4*^+^* helper T lymphocytes) regulates the expression of costimulatory molecules and the maturation of APCs (136) and triggers the process of DC-licensing. The latter empowers APCs for the activation and maintenance of cytotoxic T lymphocyte responses, increasing the levels of CD80/86 and interleukin-12 (137–139). The process also promotes B lymphocyte survival, class-switching, and antibody secretion, highlighting the role of DC-licensing in the regulation of B-cell responses in a T-cell independent way (140–142). Therefore, DC-licensing using CD40L or an anti-CD40 antibody for antigen delivery potentiates the APC to activate cytotoxic and humoral responses, independent of CD4^+^ T lymphocyte cooperation. Additionally, using mAbs as agonists to CD40 enables efficient antigen cross-presentation (143, 144).

To evaluate CD40 targeting potential, a DNA vaccine based on bovine CD154 (CD40L) fused with bovine herpesvirus 1 (BHV-1) glycoprotein D (gD) was developed. CD154-gD was capable of binding bovine and ovine lymphocytes, and thus, sheep was used as the model for *in vivo* assays. Here, the targeted group showed antigen-specific IL-4-dependent lymphocyte proliferation, increased antibody levels, and high NAb titers after boosting (51). When tested in calves, similar antibody production was observed between calves and sheep. In calves, no effect was observed when targeting the gD antigen to the CD40 receptor regarding IFN-γ secreting cells, while the nontargeted group showed increased IFN-γ secreting cells at day 8 postchallenge. Moreover, no significant differences were found in clinical signs between targeted and nontargeted calves. These studies clearly show the different responses between species when a one-size-fits-all approach is applied (53).

A DNA vaccine encoding bovine CD154 protein fused to antigens of *Toxoplasma gondii*, specifically rhoptry protein 1 (ROP1), which participates in the initial stages of invasion. The vaccine was evaluated in sheep, where a strong IgG1 response was observed after 1 week of immunization, while IgG2 values were modest. Similarly, IFN-γ levels increased significantly after the first week postimmunization compared to the nontargeted group (52). Exploiting the benefits of viral vectors and antigen targeting, Thacker et al. (57) developed adenovirus 5 (Ad5) encoding CD40L fused to tumor-associated antigens using carcinoembryonic antigen as a model to elicit an antitumoral response in dogs. The strategy resulted in the activation of T lymphocytes in 3 out of the 5 immunized dogs, although a lower anti-CEA antibody response was observed in the targeted group than in the nontargeted group (57).

In a proof-of-concept report, Chen et al. (54) evaluated the ability of a previously developed mAb, anti-chicken CD40, to induce antigen-specific antibody responses using a peptide from the ectodomain of influenza virus matrix protein 2 (M2e) as a model antigen. Four days after a single immunization, a significant increase in antigen-specific IgG antibody levels was observed in the targeted group regardless of the dose (10, 30, and 90 µg). By day 14, doses of 30 and 90 µg still presented high levels of antigen-specific antibody response in the targeted group (54). Following this, the aforementioned Me2-antigenized antibody was used to stimulate mucosal antibody responses by exploring different administration routes: cloacal drinking, oculonasal administration, and oral immunization using an alginate sphere suspension. Similar to previous findings, antigen targeting to CD40 resulted in an early antigen-specific antibody response after a single dose at 7 days postimmunization. Interestingly, all routes, including subcutaneous routes, proved capable of inducing mucosal responses, as evidenced by high IgA levels in the trachea (55). Once its capacity to induce rapid antibody production was established, this antibody served as the basis for the development of a bispecific antibody that binds CD40 and the M2e peptide of the AIV (56). The bispecific antibody would then capture the M2e^+^ viral particles in circulation and deliver them to CD40^+^ APCs, potentiating antigen uptake and response, doubling as antigen carrier and adjuvant. High hemagglutination titers were observed when applied subcutaneously, in comparison with oral and ocular-nasal routes. A prime-boost strategy using a subcutaneous route of administration was capable of inducing complete protection against lethal H5N1 highly pathogenic AIV challenge. The proposed strategy is very promising for enhancing vaccine efficacy in chickens and could be adjusted into a more cost-effective tool in the future.

### 4.2 B7 (CD80/86)

CD80 and CD86, also known as B7, are both coreceptors expressed on all APCs, such as DCs, B lymphocytes, and macrophages, and play an essential role in T-cell activation (145, 146). Their ligands are CD28, which activates T lymphocytes, and CTLA-4 (CD152), which represses cell activation (147). Thus, antigen targeting to B7 through CTLA-4 has been explored as a strategy for reaching all subpopulations of APCs.

In sheep, phospholipase D (PLD) antigen from *Corynebacterium pseudotuberculosis* was bound to bovine CTLA-4 and used to evaluate APC targeting by intramuscular DNA vaccination. When evaluating the humoral response, the total titers of PLD-specific antibodies were higher in the targeting group, allowing for enhanced clinical protection after *C. pseudotuberculosis* challenge (34). When the 45TR antigen from *Taenia ovis* was targeted using CTLA-4 in mice and sheep, an increased humoral response was observed in mice, specifically IgG1, but no positive effect on the humoral response was observed in sheep. Targeting the B7 coreceptor did not promote a protective effect against a *T. ovis* challenge in either species (35).

*Fasciola hepatica* has been described as a protozoan of importance in the livestock industry. In sheep, CatB from *F. hepatica* was targeted to APCs through CTLA-4 using a DNA prime/protein boost strategy. Immunized animals produced higher total IgG titers and lymphocyte proliferative responses than the nontargeted group (36, 42). However, when targeting the FhPGK antigen from *F. hepatica*, following a DNA prime/protein boost scheme, sheep were not protected against *F. hepatica* challenge, echoed by a failure to stimulate humoral and cellular immune responses (37).

In swine, a DNA vaccine encoding CTLA-4 and OVA as antigens augmented IgG1, IgG2, and IgA antibodies followed by 100% seroconversion after a complete immunization schedule (38). Likewise, an intradermal DNA vaccine consisting of the HANG34 peptide from SIV fused to CTLA-4 increased the total and NAbs reflected in a reduction in viral load and virus spread. However, there were no differences in pathological lesions compared with the nontargeted group (39).

Targeting GP5 protein from PRRSV via a DNA vaccine in mice favored an increase in total and NAbs along with higher IFN-γ expression in the targeted group (40). Additionally, using a tumor-induced swine model, targeting APCs using porcine CTLA-4 combined with a truncated diphtheria toxin fusion protein triggered the depletion of tumoral cells *in vivo* (43). On the other hand, targeting β-galactosidase (β-gal) from *Escherichia coli* (*E. coli*) to CTLA-4 on cattle through a DNA vaccine failed to stimulate the humoral and cellular immune response, even when trying different routes of immunization (41).

### 4.3 CD83

An early activation marker predominantly expressed in DCs and other APCs. Recent publications have just begun exploring CD83’s potential for antigen targeting. Using scFv as a delivery system, an antigenic region of the hemagglutinin protein of H9N2 (HAH9) AIV was targeted toward the avian CD83 receptor. This approach significantly increased the expression of IFN-γ, IL-6, IL-1β, IL-4, and CXCL12 in stimulated splenocytes from immunized birds. Likewise, early antibody production, virus neutralization, and hemagglutination inhibition titers were significantly enhanced by CD83 targeting. In line with this, the targeted group showed lower levels of viral shedding and high survival in challenged animals. Overall, this strategy seems to strongly induce a robust immune response capable of providing sufficient levels of protection in this model, comparable to traditional inactivated vaccines (58).

Shrestha et al. (2022) also evaluated the efficacy of the CD83 antigen-targeting strategy to circumvent the negative effects of MDA in traditional vaccines by immunizing progeny chickens after hatching (day 1 or 14). The antibody response to the targeted antigen was able to thrive with a steady and significant increase until the end of the evaluation at 84 days postvaccination; meanwhile, MDA levels started to decrease to marginal levels by days 28-35. The antibody levels and hemagglutination titers of the targeted group far surpassed those in the nontargeted group and traditional vaccine group, positioning the CD83 targeting strategy as an excellent candidate for next-generation vaccine development (59).

## 5 Targeting Dendritic cells (DC-peptides)

DC-peptides (DC-pep) are peptides obtained through phage display technologies with the ability to recognize DCs from other leucocyte populations, although their mechanism of action is unclear (148, 149). This approach has been widely studied to develop oral vaccines carried by lactic acid bacilli, thus eliciting mucosal immunity even without additional adjuvants (150). The most common bacteria used in DC-peptide targeting is *Lactobacillus plantarum*. In poultry, *L. plantarum* coated with 12-mer DC-pep and HN antigen from NDV enhanced the expression of mucosal secretory IgA (SIgA) as well as a higher frequency of splenic CD4^+^ T cells. However, the hemagglutination inhibition titers and survival postchallenge were not improved (67). Targeting *L. plantarum* with HA from AIV H9N2 enhanced the expression of activation markers such as MHC-II and CD80/86. Additionally, obtaining a robust increase in mucosal SIgA, IgG, and the expression of IFN-γ, TNF-α, IL-6, IL-10, IL-12p70, and IL-4 reduced the tissue viral load, thus allowing for better clinical protection (68, 69). When *Enterococcus faecalis* expressing DC-pep carrying the 3-1E antigen from *Eimeria tenella*, causative of avian coccidiosis, was evaluated through oral vaccination, immunized chickens presented higher IgA and IgG titers as well as a higher frequency of CD4^+^ T *cells* and expression of IFN-γ. However, the response was insufficient to provide protection after an experimental challenge (70).

*Lactobacilli* expressing DC-pep carrying different PEDV antigens, such as core neutralizing epitope (COE) or S, have been evaluated on swine DCs. The main results show enhanced activation markers such as CD80, CD86, and MHC-II on CD11c DCs and higher serum antibodies compared with the nontargeted group. In the same manner, the response of mucosal IgA was improved along with IL-4, IFN-γ, and the proliferative response (60). When evaluating the same strategy on swine, a biased reinforcement of the Th1 over Th2 profile was observed, as evidenced by a higher presence of CD4^+^IFN-γ^+^ cells than the presence of CD4^+^IL-4^+^ cells. Moreover, the probiotic/vaccine-targeted group presented a higher survival rate after a viral challenge with reduced viral load and symptom severity (61). Finally, targeting COE antigens in swine resulted in increased maturation of swine moDCs and DCs *in situ* by CD40, CD80, and CD86 expression, enhanced phagocytic activity, and TLR-2, TLR-6, and TLR-9 expression. The cellular immune response was also boosted by stimulating the expression of the Th1 cytokines IFN-γ, IL-12, and IL-17 (62). In mice, targeting *L. plantarum* DC-pep with the S antigen from PEDV enhanced the expression of CD80 in CD11c DCs and increased the titers of mucosal IgA and serum IgG along with IL-17 and IFN-γ expression. In addition, the targeting group presented higher virus neutralization up to 42 dpv (63).

Targeting E2 from BVDV in a murine model resulted in higher expression of CD40 on DCs without changes in CD86 expression. The humoral and cellular responses were significantly improved, as evidenced by a higher titer of IgG NAbs and mucosal IgA compared with the nontargeted group and lymphoproliferation in response to E2 stimuli (66). Similar results have been observed when targeting the S antigen from transmissible gastroenteritis virus (TGEV) in swine, with overexpression of the activation markers CD80/86, CD40 and MHC-II, TLR-2, and TLR-9 as well as IgG and mucosal antibodies. Additionally, the frequency of CD4 T lymphocytes IFN-γ^+^, IL-4, IL-17, IFN-γ, and TGF-β levels were increased in mucosal-associated lymph tissue (64).

On the other hand, virus-like particles (VLPs) are commonly chosen platforms for vaccine design and development. Hence, VLPs were used and coated with DC-pep, carrying HN and M antigens from NDV but also HA from AIV as a bivalent vaccine candidate. The VLP-DC-pep targeting system enhanced the expression of the activation marker MHC-II on DCs, titers of mucosal IgA, and a higher frequency of splenic CD4^+^ T cells, leading to a reduction in viral load (71).

VLPs from the PCV2 capsid carry DC-binding peptides to mouse DCs to improve both humoral and cellular immune responses. These resulted in higher activation marker expression of MHC-II, CD80, CD86, expression of IL-6, IL-10, IFN-γ lymphoproliferation, and anti-Cap IgG1 and IgG2a NAbs levels (65). In mice, targeting G antigen from rabies virus (RABV) showed a similar effect, increasing activation markers, total IgG antibodies, and both Th1 and Th2 mediated by CD4^+^ IFN-γ^+^ T and CD4^+^IL-4^+^ T cells with a skew to Th1 profile polarization. This humoral and cellular immune response provided approximately 60% of clinical protection after a viral challenge (72). It is interesting to highlight that although the targeting mechanism is not clearly defined, the approach using DC-pep targeting is undoubtedly highly efficient in promoting DC maturation, triggering the cellular response, especially Th1 cytokines, and enhancing the production of IgG and IgA antibodies. Therefore, the use of DC-pep is a promising strategy for developing new oral vaccines to control diseases affecting domestic animals by activating systemic and mucosal responses.

## 6 Targeting CD11c

The CD11c receptor belongs to the integrin family and is mostly expressed, but not restricted, by macrophages, DCs, and other myeloid cells (151, 152). CD11c is considered a DC marker in mice (153). The receptor participates in cell-to-cell adhesion but also mediates phagocytosis of extracellular material such as lipopolysaccharide, fibrinogen, collagen, etc. (154–156). In mice, CD11c is expressed at high levels on conventional DCs with the potential for cross-presentation when used in antigen targeting (157, 158). In chickens, an anti-CD11c scFv fused with the ectodomain of H9N2 influenza hemagglutinin induced prompt and effective antibody responses, with higher neutralization and hemagglutination inhibition titers than nontargeted vaccination. Additionally, CD11c targeting resulted in increased cellular responses with significantly higher cytokine production of IFN-γ, IL-6, IL-1β, and IL-4 compared to the DEC205 targeted group, which may be related to a greater expression of CD11c than DEC205 in chickens (49).

In sheep, the targeting of the Gn antigen peptide from RVFV to CD11c using a DNA vaccine resulted in poor production of antigen-specific antibodies in comparison with the nontargeted DNA vaccine group, which had higher mRNA expression levels than the targeted group. In any case, IFN-γ levels were not successfully stimulated by either DNA vaccine. Clinical scores were also lower in the nontargeted group, with CD11c targeting having almost double the score in immunized sheep (46).

SIV antigens have also been targeted to CD11c receptors using a mAb fused to target conserved antigens HA2, M2e, and NP. In this case, two routes were evaluated: intramuscular and intradermal. When applied intramuscularly, antigen targeting to porcine CD11c has been shown to significantly stimulate the IFN-γ T-cell response. Interestingly, the site of immunization appeared to have a greater effect on the elicited immune responses than the targeting itself. Intramuscular application was more effective overall, and intradermal immunization resulted in exacerbated clinical signs and viral shedding in challenged pigs, implying the significance of the delivery route along with the delivery vehicle (47). Finally, a combination of a DNA vaccine encoding a scFv anti-CD11c fused with various T-cell epitopes of PRRSV and a modified live virus (MLV) vaccine in a prime-boost strategy resulted in an increase in antigen-specific IFN-γ secreting cells (98).

## 7 Targeting sialoadhesins (Siglec and CD169)

Sialodhesin (Sn), CD169, or Siglec-1 is recognized as the sialic acid binding receptor and is well known as a highly expressed macrophage marker on tissue and secondary lymphoid organs (6, 159, 160). CD169 plays an important role in cell-to-cell adhesion and CD169^+^ macrophage-mediated antigen delivery to lymphatic resident DCs, enabling cross-presentation (161–163). CD169 macrophages by themselves cannot cross-present antigens, but they are able to transfer antigens to DCs, enabling cross-presentation (162, 164).

As an endocytic receptor in APCs, the Sn receptor has been proposed as a tool to improve antigen uptake and enhance T-cell responses. Using a mouse mAb to target porcine Sn, Revilla et al. (96) were able to induce potent T proliferative responses in IFN-α-treated monocytes and moDCs, up to 100 times more than when an irrelevant isotype control mAb was administered (96). A follow-up study by this group evaluated the proficiency of this and other mouse mAb anti-Sn to induce antigen-specific proliferation in peripheral blood mononuclear cells (PBMCs) and antibody production following a prime and boost strategy. All three of the targeting mAbs tested were capable of significantly increasing antigen-specific IgG levels in sera, with IgG1 and IgG2 profiles very similar in proportion, and once more improving proliferative responses as previously observed (50).

Likewise, antigen-specific IgG and IgM production were also observed as a response to targeting human serum album chemically linked to mAb anti-Sn receptors in the absence of adjuvants when administered in pigs. Following this study, a recombinant mAb, anti-Sn, was used to deliver PRRSV GP4 to porcine macrophages by immunizing pigs intramuscularly and challenging them seven weeks postimmunization. The strategy resulted in an increase in antigen-specific IgG and NAbs titers in sera in a dose-dependent manner, as well as rapid virus clearance (97).

## 8 Targeting chemokine receptors

### 8.1 XCR1

XCR1 is a chemokine receptor whose unique ligand is the chemokine XCL1 and specifically chemoattracts the equivalent cDC1 population in mice and humans (165, 166). In many species, this chemokine receptor is considered a conserved marker on the subset of highly efficient cross-presenting cDC1 (167–169). Therefore, targeting XCR1 seems to be a highly specific strategy to deliver antigens to the cDC1 subset.

In swine, targeting intradermal XCR1^+^ cDC1 with dimeric ligand XCL1 joined to M2e antigens from SIV resulted in higher total IgG anti-M2e antibodies. Additionally, targeting XCL1 enhances the IgG2 response in influenza-seronegative pigs and IgG1 in seropositive pigs, without a skewed effect by either CpG or MPLA adjuvants (99). Additionally, a DNA vaccine encoding the XCR1 ligand fused to B and T epitopes of the N antigen from PRRSV was used in a DNA-MLV prime-boost strategy in pigs. The DNA vaccine was combined with cationic polylactoglycolide acid (PLGA) nanoparticles. DNA vaccine alone failed to elicit humoral and cellular immune responses but, under a DNA-MLV prime-boost schedule, achieved enhancement of the anti-N IgG response (98). The authors discuss the possibility that nanoparticles affected the efficacy of the DNA vaccine and therefore, they restructured the strategy, employing naked DNA to deliver N, NSP1β, and pGP4GP5 M from PRSSV toward XCR1^+^ DC, followed by a boost with an MLV. The XCR1 targeting-MVL boost allowed for a higher S/P ratio against the N antigen at 58 dpv; nonetheless, it was not possible to find significant levels of IFN-γ secreting cells after *in vitro* restimulation or clinical protection after heterologous PRRSV challenge (48). It is important to highlight that in these last two PRRSV antigen-targeting studies, the authors used DNA vaccines without additional adjuvants or immunostimulants, which may be necessary for proper stimulation of the cellular response. In cattle, targeting XCR1 cDC1 with the XCL1 fusion protein carrying the multiepitope OB7 antigen of FMDV was applied intramuscularly alone or with oil adjuvant or poly I:C. XCR1 targeting allowed for higher total and NAbs compared to the nontargeted group, eliciting better clinical protection against viral challenges with FMDV. Interestingly, poly I:C weakened the humoral response (100).

Notably, since the cDC1 population is well known to skew toward the Th1 cytokine profile and XCR1 is highly conserved in this population, an increased cellular response would be expected as a result of XCR1 targeting (13, 170); nevertheless, this has not been evidenced by the reports mentioned above. In summary, these findings highlight the different outcomes for XCR1 targeting regarding the species, type of targeted vaccine, and type of adjuvant involved.

### 8.2 CCR1, CCR3, CCR5

Chemokine receptors, which are expressed in many cells, can effectively facilitate antigen uptake, processing, and presentation in APCs (171). In mice, targeting low immunogenic tumoral antigens to chemokine receptors successfully activated the adaptive immune response and protected against a lethal challenge without the need for adjuvants (172).

DNA vaccines containing either the gene for MIP1α chemokine, targeting CCR1/3/5 chemokine receptors or a scFv anti-MHC-II along with fused HA antigen of H7N1 AIV were developed to stimulate APC-specific responses. When tested in mice, CCR1/3/5 and MHC-II targeting resulted in slightly higher IFN-γ T-cell responses than CCR1/3/5 targeting (45).

A similar approach was evaluated in pigs using a DNA vaccine encoding the MIP1α chemokine fused to HA antigen from the H1N1 influenza virus. The antibody response favored the IgG2 isotype over IgG1, while virus neutralization titers appeared higher in the CCR1/3/5 targeted group than in the antigen-only group. In addition, T-cell responses were significantly enhanced in the targeted group in a cross-reactive manner, responding to H1, H5, and H13 influenza subtypes, 28 days postimmunization (44). These findings suggest a notably efficient cellular immune response elicited in pigs by this targeting strategy.

## 9 Others

### 9.1 CD163 (scavenger receptor)

The scavenger receptor, also known as the CD163 receptor, contains nine scavenger cysteine-rich domains and is restricted to cells of the monocytic lineage (173). It is expressed at high levels in mature macrophages and low levels in moDCs (174, 175). This endocytic receptor has been characterized in several species, although there is no evidence that targeting CD163 can allow for cross-presentation (176). Although CD163 has been widely studied concerning its participation in infectious diseases in pigs, its antigen-targeting potential has not been equally explored. A report from Poderoso et al. (50) showed that targeting mouse IgG as an immunogen to CD163 following a prime-boost strategy resulted in the stimulation of the proliferative response in PBMCs. Additionally, the humoral response was greatly enhanced compared to the isotype control as early as 2 weeks postimmunization and increased with a booster dose at 6 weeks post-priming. This humoral response was particularly skewed to the IgG2 subclass and remained significantly higher than the negative control until 17 weeks after immunization (50).

### 9.2 Fcγ-receptor

Fc-γ receptors (Fc-γR) are distributed ubiquitously in endothelial, myeloid, and lymphoid cells and perform an essential function in the immune system by recognizing antigen-antibody complexes, thus improving antigen capture and processing (177–179). It is well known that antigens fused to IgG-Fc domains significantly enhance the immunogenicity of the antigen due to increased uptake through Fc-g receptors (180–182).

*In vitro* studies have evaluated the potential of using Fc receptors for antigen targeting by using porcine moDCs. Immuno-complexes (ICs) composed of F4 fimbriae from enterotoxigenic *E. coli* and anti-F4 polyclonal antibodies were incubated with porcine monocytes and respective cytokines to stimulate the generation of moDCs. F4-IC was internalized and enhanced the upregulation of the DC activation markers MHC-II, CD40, and CD80/86. Subsequently, activated moDCs could induce robust lymphocyte proliferation compared with F4 antigen- or IgG-only treated moDCs. Moreover, stimulated moDCs enhanced their production of IL-1β, IL-6, IL-8, and TNF-α, similar to a flagellin control but higher than F4 antigen or IgG-only treated moDCs. These findings demonstrate the maturation of moDCs induced by targeting Fc receptors and their potential use in antigen-targeting-based vaccines (81).

### 9.3 M-cells

In hopes of enhancing mucosal immune responses against pathogens, M-cell targeting was evaluated using a targeted unit named ligand Co-1 coupled with TB1 protein of FMDV and displayed in *Lactococcus lactis* (*L. lactis*-TB1-Co1) for increased stimulation. Mice and guinea pigs were orally immunized with *L. lactis*-TB1-Co1 and subsequently challenged 30 days postvaccination. In mice, the results showed increased antigen-specific IgA levels in sera, intestinal, and lung lavage fluids in the targeted group in comparison to the nontargeted and inactivated vaccine groups. Regarding cellular immune stimulation, mice in the targeted group presented higher T-cell proliferation and appeared to have enhanced IFN-γ and IL-2 production than the nontargeted group. The effect on humoral and cellular immune responses in guinea pigs was not as evident as in mice, although 60% protection was observed when animals were challenged. Once more, these findings highlight the different responses between species to a single targeting strategy (84).

## 10 Conclusions and future directions

The use of antigen-targeting strategies in the field of veterinary medicine has been evaluated in several species; swine is the most scrutinized specie, followed by chickens, cattle, and sheep. The available information shows highly heterogeneous responses according to the type of APC receptors targeted pertaining to humoral, cellular, and clinical protection. Most studies that determined clinical protection were evaluated under controlled experimental conditions; however, their efficacy under field conditions remains unknown. Remarkably, among all the vaccination routes evaluated in antigen targeting, oral vaccination with DC-pep-expressing lactobacillus seems to be a very promising strategy, showing high consistency in the induction of both mucosal and systemic responses. On the other hand, parenteral targeting with MHC-II-DR has also been widely successful, culminating in the approval of a commercial vaccine in South America.

It is important to continue with the development and evaluation of APC-targeting vaccines and generate knowledge that undoubtedly could help to modify conditions to redefine current vaccine trends and improve animal health. It is important to explore several routes of administration, antigens, and adjuvants since a one-size-fits-all strategy is very unlikely to work for all species and diseases. When the target receptor is mainly expressed on DCs, the intradermal or dermal route must be elected over the intramuscular route. Additionally, it is important to explore the nasal or intrauterine route due to the abundance of DCs in these sites. In this line, when the target receptor is mainly expressed on macrophages, the intramuscular route or oral route could be priorities. It is also important to keep in mind the use of new technologies, such as mRNA. This technology could be an interesting option to improve the benefits of antigen targeting for the control of diseases affecting veterinary medicine.

## Author contributions

Conceptualization, JH, EM-G and LB-C. Data curation, EM-G and LB-C. Writing—original draft preparation, EM-G and LB-C. Writing—review and editing, JH, EM-G and LB-C. Supervision, JH. funding acquisition, JH. All authors contributed to the article and approved the submitted version.
