# Corrigendum: Recent advances in antigen targeting to antigen-presenting cells in veterinary medicine

**DOI:** 10.3389/fimmu.2023.1188815

**Published:** 2023-06-07

**Authors:** Edgar Alonso Melgoza-González, Lorena Bustamante-Córdova, Jesús Hernández

**Affiliations:** Laboratorio de Inmunología, Centro de Investigación en Alimentación y Desarrollo A. C., Hermosillo, Mexico

**Keywords:** antigen target, antigen presenting cell, receptors, veterinary, vaccines

In the published article, there was an error in **Figure 1** as published. We noticed an error in **Figure 1** related to the structure of one of the receptors (Langerin). The corrected **Figure 1** and its caption appear below.

**Figure 1:**
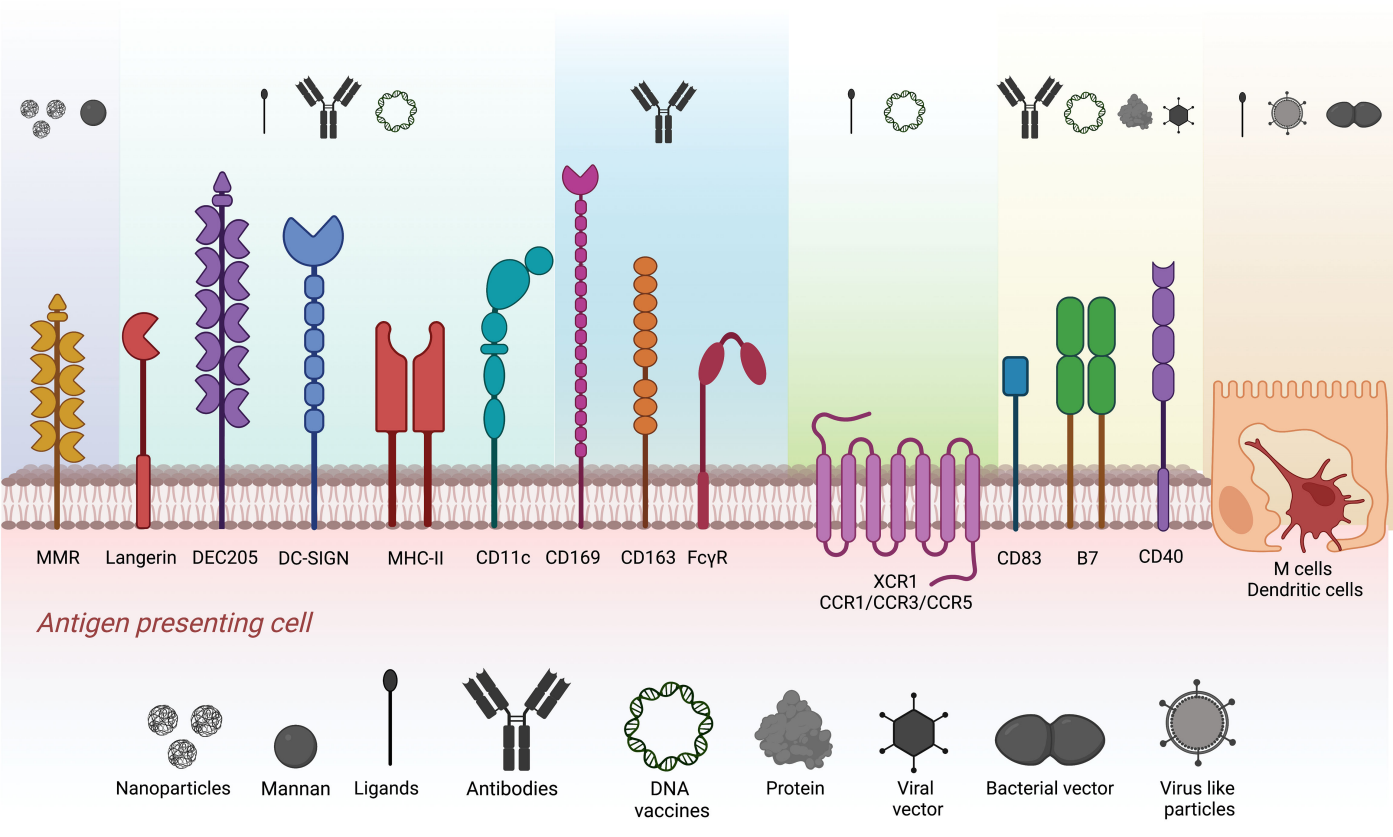
Strategies explored for antigen targeting to different APC populations. Target surface receptors on APCs and M cells evaluated in antigen targeting. Different colors represent the clusters of carriers such as nanoparticles, mannan, ligands, antibodies, DNA vaccines, proteins, virus-like particles, and viral and bacterial vectors used to target specific surface receptors.

Strategies explored for antigen targeting to different APC populations. Target surface receptors on APCs and M cells evaluated in antigen targeting. Different colors represent the clusters of carriers such as nanoparticles, mannan, ligands, antibodies, DNA vaccines, proteins, virus-like particles, and viral and bacterial vectors used to target specific surface receptors.

The authors apologize for this error and state that this does not change the scientific conclusions of the article in any way. The original article has been updated.

