# Supplementary material for: Recent advances in antigen targeting to antigen-presenting cells in veterinary medicine
Source: Front Immunol. 2023 Mar 10;14:1080238. doi: 10.3389/fimmu.2023.1080238 (PMC10038197; doi:10.3389/fimmu.2023.1080238)
Supplement: Supplementary file 1 [file Table_1.docx]

Supplementary Table 1

| Target | Specie | Antigen | Strategy | Route | Main response | Ref |
| --- | --- | --- | --- | --- | --- | --- |
| B7  (CD80/86) | Sheep/Mice | 45W of  *Taenia ovis* | DNA vaccine | IM | Higher total and IgG1 antibody production in mice albeit no effect was achieved on sheep. | (1) |
|  | Sheep | PLD of Corynebacterium pseudotuberculosis | DNA vaccine | IM | Enhanced humoral response and clinical protection against a Corynebacterium pseudo-tuberculosis challenge. | (2) |
|  | Swine | Ovoalbumin | DNA vaccine | Gene gun | Higher IgG1, IgG2, and IgA response in serum and 100% of seroconversion after a complete the scheme. | (3) |
|  | Cattle | β-gal of  *Escherichia coli* | DNA vaccine | IM, ID, Intramammary gland  Intrasupramammary lymph node. | No significant effect in both humoral and cellular immune response. | (4) |
|  | Sheep | Cat B of Fasciola hepatica | DNA vaccine | IM | Higher total antigen-specific IgG levels and lymphoproliferative response. | (5) |
|  | Cattle/Sheep  Evaluated in mice | Cat B of Fasciola hepatica | DNA vaccine | IM | Increased IgG production albeit without a positive effect in the cellular response. | (6) |
|  | Mice/Swine | GP5 of PRRSV | DNA vaccine | IM | Improved humoral and cellular response when used as a prime-boost strategy followed by a killed vaccine. | (7) |
|  | Sheep | FhPGK of *Fasciola hepatica* | DNA vaccine | IM | No significant effect. | (8) |
|  | Swine | HA of SIV | DNA vaccine  Adj: Diluvac forte MSD | ID | Increased IgG titers in sera. Reduced viral RNA in BALF. | (9) |
|  | Mice | Truncated diphtheria toxin | Protein | IP | Depletion of tumor cells and prolonged survival by toxin targeting *in vivo*. | (10) |
| CCR1/3/5 | Swine | HA of IV | DNA vaccine | ID | Cross-reactive IFN-γ T cell response. Favored IgG2 response. | (11) |
|  | Chickens | HA of AIV | DNA vaccine | ID, IP, IM | Enhanced IFN-γ T cell response. | (12) |
| CD11c | Chickens | HA of AIV | Antibody  Adj: MontanideTM ISA 71R VG | SC | Rapid antibody production, high virus neutralization and hemagglutination inhibition titers. Increased production of IFN-γ, IL-6, IL-1ꞵ and IL-4. | (13) |
|  | Sheep | Gn antigen RVFV | DNA vaccine  Adj: pGM-CSF | ID | Poor antibody and IFN-γ T cell responses. | (14) |
|  | Swine | PRRSV antigens | DNA vaccine | ID | Enhanced IFN-γ T cell and antibody responses when used in combination with a modified live virus vaccine. | (15) |
|  | Swine | HA of SIV | Antibody  Adj: CpG | ID  IM | Better IFN-γ T cell response using intramuscular targeting. Intradermal targeting resulted in exacerbated disease. | (16) |
| CD163 | Swine | Mouse Ig | Antibody  Adj: Freund (compl/incomp) | SC  IM | Enhanced PBMC proliferation and IgG response, especially IgG2. | (17) |
| CD40 | Sheep | gD of BHV-1 | DNA vaccine | ID | Lymphocyte proliferation, increased antibody production and neutralization titers. | (18) |
|  | Calves | gD of BHV-1 | DNA vaccine | ID | Increased IgG and IgA levels compared to the non-vaccinated group. No effect in IFN-γ secreting cells. | (19) |
|  | Dog | CEA | Adenovirus | ID | Higher lymphocyte proliferation and low IgG1 and IgG2 responses compared to the non-targeted group. | (20) |
|  | Sheep | ROP1 of *Toxoplasma gondii* | DNA vaccine | IM | Enhanced IgG1and IFN-γ production. | (21) |
|  | Chickens | Biotin-SA | Antibody | SC | Significant antigen-specific IgG response in targeted group. | (22) |
|  | Chickens | Biotin-SA | Antibody | SC, bursal, oral, oculo-nasal. | Increased IgA levels at 7 days after immunization. | (23) |
|  | Chickens | M2e of AIV | Antibody | SC, oral, oculo-nasal. | Protection against highly pathogenic avian influenza challenge after prime-boost. High hemagglutination inhibition titers. | (24) |
| CD83 | Chickens | HA of AIV | Antibody  Adj: Montanide ISA 71 R VG | SC | Enhanced antibody and cytokine production. Confers good levels of protection. | (25) |
|  | Chickens | HA of AIV | Antibody | SC | High antibody levels and hemagglutination inhibition titers. Not hindered by maternally derived antibodies. | (26) |
| Dendritic Cells | Chickens | HN of NDV | Bacterial vector  DC-pep | Oral | Increased levels of SIgA against NDV HN in intestine as well as higher percentage of CD4^+^ T cells both in blood and spleen. | (27) |
|  | Swine/mice | COE of PEDV | Bacterial vector  DC-pep | Oral | Enhanced expression of CD80, CD86, MHC-II on CD11c cells, total IgG, NAbs and mucosal SIgAs. Lymphoproliferation, IFN-γ and IL-4 was higher in the targeted group. | (28) |
|  | Swine | S antigen PEDV | Bacterial vector  DC-pep | Oral | Increased DC CD80^+^ expression and IL-17 and IFN-γ production. Enhanced NAbs and mucosal IgA response. | (29) |
|  | Chickens/mice | AIV | Bacterial vector  DC-pep | Oral | Higher activation markers MHC-II and CD86. Enhanced expression of IFN-γ, TNF-α, IL-6, IL-10, and IL-12p70 and mucosal SIgA response. | (30) |
|  | Swine | PEDV | Bacterial vector  DC-pep | Oral | Enhanced mucosal IgA and Th1 CD4^+^ IFN-γ^+^ responses. Higher survival and protection efficacy. Reduced severity of infection. | (31) |
|  | Cattle/mice | E2 of BVDV | Bacterial vector DC-pep | Oral | Increased levels of IgG, mucosal IgA, neutralizing activity, expression of CD40 and MHC-II, and lymphoproliferation. | (32) |
|  | Chickens | *Eimeria* target protein | Bacterial vector  DC-pep | Oral | Higher IgG and IgA levels 4 weeks post-vaccination. Enhanced CD4^+^ IFN-γ. No effect on protection when challenged. | (33) |
|  | Chickens | HN of NDV  HA of AIV | Virus like particles  DC-pep | IM  IN | Enhanced MHC-II expression. Higher mucosal IgA and spleen T CD4^+^ frequency. Lower virus shedding after challenge. | (34) |
|  | Swine/mice | Cap protein of PCV2 | Virus like particles  DC-pep  Adj: PCV2 Cap | SC | Increment in MHC-II, CD80/86, and IL-6 expression on murine DCs. Improved levels of IgG1, IgG2, Nabs, lymphoproliferation, IL-10, and IFN-γ. | (35) |
|  | Swine | COE of PEDV | Bacterial vector  DC-pep | *In vitro* | Increased expression of CD40, CD80/86, TLR2, TLR-6, TLR-9, IFN-γ, IL-12, and IL-17. Higher T cell lymphoproliferation response with a Th1 profile. | (36) |
|  | Mice | G or RABV | Bacterial vector  DC-pep | Oral | Higher expression of CD80/86 on CD11c DCs and total IgG antibodies. Skewed Th1 profile by T CD4^+^ IFN-γ^+^. Around 60% of clinical protection after a viral challenge. | (37) |
|  | Swine | S of TGEV | Bacterial vector  DC-pep | Oral | Increased TLR-2, TLR-9, CD80/86, CD40, MHC-II, serum IgG and SIgA. Higher T CD4^+^, IL-4, IL-17, IFN-γ, and TGF-β levels. | (38) |
|  | Chicken | HA of AIV | Bacterial vector  DC-pep | Oral | Higher T lymphocyte proliferation as well as IFN-γ and IL-4. Increased IgA and IgG levels with clinical protection and pulmonal pathology reduction after viral challenge. | (39) |
| DC-SIGN | Swine | PRRSV antigens | Antibody  Adj: MPLA | TD | Increased frequency of CD4^+^ IFN-γ, CD8^+^ IFN-γ and CD4^+^ CD8^+^ IFN-γ secreting T cells at 7- and 14-days post-immunization. | (40) |
|  | Swine | PRRSV antigens | Antibody  Adj: Poly I:C | IM | Increased CD4^+^ CD8^+^ IFN-γ, CD4^+^ CD8^+^ IL-4 and CD8^+^ IL-4 secreting cells at 42 days post-immunization. | (41) |
| DEC205 | Calves | MSP1 of *Anaplasma marginale* | DNA vaccine  Adj: CD40L, pFTL3L and pGM-CSF | ID | Improved CD4^+^ IFN-γ^+^ T cells, proliferation of CD4^+^ T cells, and antibody responses. | (42) |
|  | Chickens | HA of AIV | Antibody  Adj: LPS | SC | Higher and faster antibody response in the targeting group 14 days after priming. | (43) |
|  | Chickens | RSV | Antibody  Adj: Poly I:C | SC | Higher levels of IL-12, IL-2, and IFN-γ from Th1 profile after in vitro stimulation in PBMCs. | (44) |
|  | Chickens | HN of AIV | Antibody  Adj: LPS | SC | Higher titers of total and antigen-specific NAbs in the DEC205-targeted group. | (45) |
|  | Swine | PRRSV antigens | Antibody  Adj: Poly I:C | ID | Enhanced levels of anti-PRRSV Abs.  No difference in IFN-γ secreting T cells. | (46) |
|  | Sheep | Gn ectodomain of RVFV | DNA vaccine  Adj: plasmidic GM-CSF | ID | Increased frequency of T cells IFN-γ^+^.  Poor humoral response in targeted group. | (14) |
|  | Swine | Cap of PCV2 | Antibody  Adj: Poly I:C | ID | Efficient targeting cDC1 cells *in vivo*. Higher induction of CD4^+^ CD8^+^ IFN-γ^+^ T Cells.  Poor humoral response. | (47) |
|  | Chickens | HA of AIV | Antibody  Adj: MontanideTM ISA 71R VG | SC | Higher hemagglutinin-inhibition (HI) titer levels and early increase of IgM and IgY. | (13) |
|  | Swine | PRRSV antigens | Antibody  Adj: Poly I:C | IM | No positive effect in humoral and cellular response in the DEC205 targeted group. | (41) |
|  | Sheep/mice | Gn ectodomain of RVFV | DNA vaccine  Adj: pGM-CSF | ID | Higher humoral and cellular immune response in non-targeted group. | (48) |
| Fcγ receptor | Swine | F4 fimbriae of ETEC | Antibody | *In vitro* | Upregulation of MCHII, CD40 and CD80/86. Enhanced T cell proliferation and pro-inflammatory cytokines. | (49) |
| Langerin | Swine | PEDV antigens | Antibody  Adj: Cholera toxin | ID  IM | Enhanced Ag-specific T cell proliferation in the ID targeted group. IgG and IgA responses improved in IM targeted group. | (50) |
|  | Swine | PEDV antigens | Antibody  Not specified adjuvant | IM | Early cellular response in the targeted group, IL-4 and IFN-γ secreting T cells. | (51) |
|  | Swine | PRSSV antigens | Antibody  Adj: Poly I:C | IM | Slightly increased CD8^+^ IL-4-secreting cells. No positive effect in viremia after PRRSV challenge. | (41) |
| M cells | Mice/Guinea pig | TB1 protein of FMDV | Ligand  Adj: CpG | Oral  IM | Oral immunization enhanced mucosal and systemic humoral response in mice. Partial protection against FMDV in guinea pigs. | (52) |
| Mannose receptor | Swine | PCV2 antigens | Mannan | ID | Similar stimulation of IgG, IL-4, IL-2, IFN-γ as the commercial PCV2 vaccine. | (53) |
|  | Swine | PRRSV | Mannosylated gelatin NPs | *In vitro* | Enhanced activation of T cells and differentiation to effector cytotoxic T cells. | (54) |
|  | Swine | SIV antigens | Mannosylated chitosan-based NPs | IN | Increased cross-reactive IgA response. Enhanced expression of IL-4 and IFN-γ. | (55) |
| MHC-II | Dog/Rabbit | RHDV | Antibody | IM | Higher humoral response in targeted group. Full protection in rabbits after challenge. | (56) |
|  | Guinea pigs, cattle | E2 of BVDV | Antibody  Adj: Oil (Marcol Arlace) | IM | Increased NAbs response and clinical protection against challenge. | (57) |
|  | Guinea pigs and Cattle | BVDV antigens | Antibody  Adj: Montanide ISA 50 | IM | Higher Nabs in guinea pigs even at lower doses. In cattle, long-term NAbs response similar to commercial inactivated vaccine. | (58) |
|  | Guinea pigs/Cattle | E2 of BVDV | Antibody  Adj: Water-in-oil | SC or IM | Induction of NAbs in guinea pigs. In cattle, under field conditions, the targeting triggers a potent and longer NAbs and Ab response. | (59) |
|  | Guinea pigs/Cattle | VP2 of BTV | Antibody  Adj: Montanide ISA50 | IM | Similar induction of NAbs when 25% less antigen is targeted to MHC-II compared with non-targeted group. | (60) |
|  | Swine | FMDV antigens | DNA vaccine | IM | Induction of IFN-γ-secreting cells albeit no humoral response was achieved. Partial to full protection after FMDV challenge in pigs. | (61) |
|  | Swine | ASFV antigens | DNA vaccine | IM and SC | Enhanced humoral response, IFN-γ-secreting cells, and proliferation of T CD4^+^ cells. No protection against challenge. | (62) |
|  | Ferrets  Swine | HA of IV | DNA vaccine | ID | In both ferrets and pigs, single dose of vaccine enhanced total and NAbs levels. | (63) |
|  | Cattle | MSP1 of *Anaplasma marginale* | DNA vaccine  Adj: FLT3L and GM-CSF | ID | Enhanced CD4^+^ T cell proliferation response, IFN-γ production, and higher IgG levels with a fast and robust re-call response. | (64) |
| Sialoadhesin | Swine | Mouse Ig | Antibody  Adj: Freund (compl/incomp) | IM | Increased cellular response, high T cell proliferation. | (65) |
|  | Swine | Mouse Ig | Antibody  Adj: Freund (compl/incomp) | SC  IM | Enhanced PBMC proliferation and IgG response. | (17) |
|  | Swine | PRRSV | Antibody | IM | Early antibody response after challenge, compared to the control group. Slightly lower viral titers. | (66) |
| XCR1 | Swine | M2e of SIV | Ligand  Adj: MPLA or CpG | ID | Total IgG anti-M2e antibodies were higher at 28 and 56 dpv. Higher IgG2 response in influenza seronegative pigs and IgG1 in seropositive pigs. | (67) |
|  | Cattle | FMDV antigens | Ligand  Adj: Poly I:C and Montanide 201 oil | IM | Enhancement of total and Nabs. Clinical protection against challenge.  Poly I:C impaired the humoral response. | (68) |
|  | Swine | PRRSV antigens | DNA vaccine  Adj: PLGA NPs | ID | Higher levels of total serum and mucosal IgG antibodies anti-N. Inefficient stimulation of the IFN-γ response. | (15) |
|  | Swine | PRRSV antigens | DNA vaccine | ID | Increased humoral response against N antigen. No effect on IFN-γ secreting cells nor clinical protection after challenge. | (69) |

Abbreviations: IM (Intramuscular); IN (Intranasal); ID (Intradermally); SC (Subcutaneous); IP (Intraperitoneal); TD (Trasdermally); BVDV (Bovine Viral Diarrhea Virus); FMDV (Foot and Mouth Disease Virus); RHDV (Rabbit Hemorrhagic Disease Virus); ASFV (African Swine Fever Virus); PRRSV (Porcine Respiratory and Reproductive Syndrome Virus); IV (Influenza Virus); AIV (Avian Influenza Virus); BTV (Bluetongue Virus); MPLA (Mono-Phosphoryl Lipid A); PBMC (Peripheral Blood Mononuclear Cells); HA (Hemagglutinin); RVFV (Rift Valley Fever Virus); SIV (Swine Influenza Virus); gD (glycoprotein D); BHV-1 (Bovine Herpes Virus); ROP1 (Rhoptry Protein 1); CEA (Carcinoembryonic Antigen); HN (Hemagglutinin-Neuraminidase);  NDV (Newcastle Disease Virus); COE (CO-26K-equivalent Epitope); PEDV (Porcine Epidemic Diarrhea Virus); PLGA (poly(lactic-co-glycolic acid); RSV (Rous Sarcoma Virus); ETEC (Enterotoxigenic *Escherichia coli*);  OVA (Ovalbumin); HSA (Human Serum Albumin); RABV (Rabies Virus); TGEV (Transmissible Gastroenteritis Coronavirus); FhPGK (Phosphoglycerate Kinase of *Fasciola hepatica*); Cat B (Cathepsin B); β-gal (β-galactosidase); PLD (Phospholipase D); Gn (Glycoprotein n); GP5 (Glycoprotein 5); S (Spike); E2 (Glycoprotein E2); Cap (Capsid); PCV2 (Porcine Circovirus type 2); G (Glycoprotein); MSP1 (Merozoite Surface Protein-1); TB1 (multiepitope); VP2 (Outer capsid protein).

1. Drew DR, Boyle J, Lew A, Lightowlers M, Chaplin P, Strugnell R. The comparative efficacy of CTLA-4 and L-selectin targeted DNA vaccines in mice and sheep. Vaccine. 2001;19(31):4417-28.

2. Chaplin PJ, De Rose R, Boyle JS, McWaters P, Kelly J, Tennent JM, et al. Targeting improves the efficacy of a DNA vaccine against Corynebacterium pseudotuberculosis in sheep. Infection and immunity. 1999;67(12):6434-8.

3. Tachedjian M, Boyle JS, Lew AM, Horvatic B, Scheerlinck J-PY, Tennent JM, et al. Gene gun immunization in a preclinical model is enhanced by B7 targeting. Vaccine. 2003;21(21-22):2900-5.

4. Shkreta L, Talbot BG, Lacasse P. Optimization of DNA vaccination immune responses in dairy cows: effect of injection site and the targeting efficacy of antigen-bCTLA-4 complex. Vaccine. 2003;21(19-20):2372-82.

5. Kennedy NJ, Spithill TW, Tennent J, Wood PR, Piedrafita D. DNA vaccines in sheep: CTLA-4 mediated targeting and CpG motifs enhance immunogenicity in a DNA prime/protein boost strategy. Vaccine. 2006;24(7):970-9.

6. Jayaraj R, Piedrafita D, Spithill T, Smooker P. Evaluation of the immune responses induced by four targeted DNA vaccines encoding the juvenile liver fluke antigen, cathepsin B in a mouse model. Genetic Vaccines and Therapy. 2012;10(1):1-9.

7. Wang Y, Zhao H, Ma Z, Wang Y, Feng W-h. CTLA4 mediated targeting enhances immunogenicity against PRRSV in a DNA prime/killed virus boost strategy. Veterinary immunology and immunopathology. 2013;154(3-4):121-8.

8. Wesołowska A, Basałaj K, Zawistowska-Deniziak A, Januszkiewicz K, Ljunggren MK, Jedlina L, et al. The failure of a DNA prime/protein boost regime and CTLA-4 mediated targeting to improve the potency of a DNA vaccine encoding Fasciola hepatica phosphoglycerate kinase in sheep. Veterinary immunology and immunopathology. 2019;217:109941.

9. Sisteré-Oró M, Vergara-Alert J, Stratmann T, López-Serrano S, Pina-Pedrero S, Córdoba L, et al. Conserved HA-peptide NG34 formulated in pCMV-CTLA4-Ig reduces viral shedding in pigs after a heterosubtypic influenza virus SwH3N2 challenge. PloS one. 2019;14(3):e0212431.

10. Peraino JS, Schenk M, Zhang H, Li G, Hermanrud CE, Neville Jr DM, et al. A truncated diphtheria toxin based recombinant porcine CTLA-4 fusion toxin. Journal of immunological methods. 2013;391(1-2):103-11.

11. Grodeland G, Fossum E, Bogen B. Targeting of HA to chemokine receptors induces strong and cross-reactive T cell responses after DNA vaccination in pigs. Vaccine. 2020;38(6):1280-5.

12. Andersen TK, Zhou F, Cox R, Bogen B, Grødeland G. A DNA vaccine that targets hemagglutinin to antigen-presenting cells protects mice against H7 influenza. Journal of virology. 2017;91(23):e01340-17.

13. Shrestha A, Sadeyen J-R, Lukosaityte D, Chang P, Van Hulten M, Iqbal M. Targeting Haemagglutinin Antigen of Avian Influenza Virus to Chicken Immune Cell Receptors Dec205 and CD11c Induces Differential Immune-Potentiating Responses. Vaccines. 2021;9(7):784.

14. Chrun T, Lacôte S, Urien C, Jouneau L, Barc C, Bouguyon E, et al. A Rift Valley fever virus Gn ectodomain-based DNA vaccine induces a partial protection not improved by APC targeting. npj Vaccines. 2018;3(1):1-13.

15. Bernelin-Cottet C, Urien C, Stubsrud E, Jakob V, Bouguyon E, Bordet E, et al. A DNA-modified live vaccine prime–boost strategy broadens the t-cell response and enhances the antibody response against the porcine reproductive and respiratory syndrome virus. Viruses. 2019;11(6):551.

16. Bernelin-Cottet C, Deloizy C, Stanek O, Barc C, Bouguyon E, Urien C, et al. A universal influenza vaccine can lead to disease exacerbation or viral control depending on delivery strategies. Frontiers in immunology. 2016;7:641.

17. Poderoso T, Martínez P, Álvarez B, Handler A, Moreno S, Alonso F, et al. Delivery of antigen to sialoadhesin or CD163 improves the specific immune response in pigs. Vaccine. 2011;29(29-30):4813-20.

18. Manoj S, Griebel PJ, Babiuk LA. Targeting with bovine CD154 enhances humoral immune responses induced by a DNA vaccine in sheep. The Journal of Immunology. 2003;170(2):989-96.

19. Manoj S, Griebel PJ, Babiuk LA, Van Drunen Littel‐Van Den Hurk S. Modulation of immune responses to bovine herpesvirus‐1 in cattle by immunization with a DNA vaccine encoding glycoprotein D as a fusion protein with bovine CD154. Immunology. 2004;112(2):328-38.

20. Thacker EE, Nakayama M, Smith BF, Bird RC, Muminova Z, Strong TV, et al. A genetically engineered adenovirus vector targeted to CD40 mediates transduction of canine dendritic cells and promotes antigen-specific immune responses in vivo. Vaccine. 2009;27(50):7116-24.

21. Hiszczyńska-Sawicka E, Li H, Xu JB, Holec-Gąsior L, Kur J, Sedcole R, et al. Modulation of immune response to Toxoplasma gondii in sheep by immunization with a DNA vaccine encoding ROP1 antigen as a fusion protein with ovine CD154. Veterinary Parasitology. 2011;183(1-2):72-8.

22. Chen C-H, Abi-Ghanem D, Waghela SD, Chou W-K, Farnell MB, Mwangi W, et al. Immunization of chickens with an agonistic monoclonal anti-chicken CD40 antibody–hapten complex: Rapid and robust IgG response induced by a single subcutaneous injection. Journal of immunological methods. 2012;378(1-2):116-20.

23. Chou W-K, Chen C-H, Vuong CN, Abi-Ghanem D, Waghela SD, Mwangi W, et al. Significant mucosal sIgA production after a single oral or parenteral administration using in vivo CD40 targeting in the chicken. Research in Veterinary Science. 2016;108:112-5.

24. Vuong CN, Chou W-K, Briggs W, Faulkner O, Wolfenden A, Jonas M, et al. Crude Inactivated Influenza A Virus Adjuvated with a Bispecific Antibody Complex Targeting Chicken CD40 and AIV M2e Confers Protection Against Lethal HPA I Challenge in Chickens. Monoclonal Antibodies in Immunodiagnosis and Immunotherapy. 2018;37(6):245-51.

25. Shrestha A, Sadeyen J-R, Lukosaityte D, Chang P, Smith A, Van Hulten M, et al. Selectively targeting haemagglutinin antigen to chicken CD83 receptor induces faster and stronger immunity against avian influenza. npj Vaccines. 2021;6(1):1-12.

26. Shrestha A, Meeuws R, Sadeyen J-R, Chang P, Van Hulten M, Iqbal M. Haemagglutinin antigen selectively targeted to chicken CD83 overcomes interference from maternally derived antibodies in chickens. NPJ vaccines. 2022;7(1):1-8.

27. Jiang Y, Hu J, Guo Y, Yang W, Ye L, Shi C, et al. Construction and immunological evaluation of recombinant Lactobacillus plantarum expressing HN of Newcastle disease virus and DC-targeting peptide fusion protein. Journal of biotechnology. 2015;216:82-9.

28. Wang X, Wang L, Huang X, Ma S, Yu M, Shi W, et al. Oral delivery of probiotics expressing dendritic cell-targeting peptide fused with porcine epidemic diarrhea virus COE antigen: a promising vaccine strategy against PEDV. Viruses. 2017;9(11):312.

29. Huang K-Y, Yang G-L, Jin Y-B, Liu J, Chen H-L, Wang P-B, et al. Construction and immunogenicity analysis of Lactobacillus plantarum expressing a porcine epidemic diarrhea virus S gene fused to a DC-targeting peptide. Virus research. 2018;247:84-93.

30. Sun Y, Qian J, Xu X, Tang Y, Xu W, Yang W, et al. Dendritic cell-targeted recombinantLactobacilli induce DC activation and elicit specific immune responses against G57 genotype of avian H9N2 influenza virus infection. Veterinary microbiology. 2018;223:9-20.

31. Hou X, Jiang X, Jiang Y, Tang L, Xu Y, Qiao X, et al. Oral immunization against PEDV with recombinant Lactobacillus casei expressing dendritic cell-targeting peptide fusing COE protein of PEDV in piglets. Viruses. 2018;10(3):106.

32. Wang Y, Feng B, Niu C, Jia S, Sun C, Wang Z, et al. Dendritic cell targeting of bovine viral diarrhea virus E2 protein expressed by Lactobacillus casei effectively induces antigen-specific immune responses via oral vaccination. Viruses. 2019;11(6):575.

33. Chen W, Ma C, Wang D, Li G, Ma D. Immune response and protective efficacy of recombinant Enterococcus faecalis displaying dendritic cell–targeting peptide fused with Eimeria tenella 3-1E protein. Poultry science. 2020;99(6):2967-75.

34. Xu X, Qian J, Qin L, Li J, Xue C, Ding J, et al. Chimeric Newcastle disease virus-like particles containing DC-binding peptide-fused haemagglutinin protect chickens from virulent Newcastle disease virus and H9N2 avian influenza virus challenge. Virologica Sinica. 2020;35(4):455-67.

35. Lu Y, Liu Z-H, Li Y-X, Xu H-L, Fang W-H, He F. Targeted Delivery of Nanovaccine to Dendritic Cells via DC-Binding Peptides Induces Potent Antiviral Immunity in vivo. International Journal of Nanomedicine. 2022;17:1593.

36. Xia T, Yang H, Guo Y, Guo T, Xin L, Jiang Y, et al. Human dendritic cell targeting peptide can be targeted to porcine dendritic cells to improve antigen capture efficiency to stimulate stronger immune response. Frontiers in Immunology. 2022;13.

37. Wang J, Jiang H, Yang R, Zhang S, Zhao W, Hu J, et al. Construction and evaluation of recombinant Lactobacillus plantarum NC8 delivering one single or two copies of G protein fused with a DC-targeting peptide (DCpep) as novel oral rabies vaccine. Veterinary Microbiology. 2020;251:108906.

38. Jin Y-B, Yang W-T, Shi C-W, Feng B, Huang K-Y, Zhao G-X, et al. Immune responses induced by recombinant Lactobacillus plantarum expressing the spike protein derived from transmissible gastroenteritis virus in piglets. Applied microbiology and biotechnology. 2018;102(19):8403-17.

39. Shi S-H, Yang W-T, Yang G-L, Zhang X-K, Liu Y-Y, Zhang L-J, et al. Lactobacillus plantarum vaccine vector expressing hemagglutinin provides protection against H9N2 challenge infection. Virus research. 2016;211:46-57.

40. Subramaniam S, Piñeyro P, Tian D, Overend C, Yugo DM, Matzinger SR, et al. In vivo targeting of porcine reproductive and respiratory syndrome virus antigen through porcine DC-SIGN to dendritic cells elicits antigen-specific CD4T cell immunity in pigs. Vaccine. 2014;32(50):6768-75.

41. Subramaniam S, Piñeyro P, Derscheid RJ, Madson DM, Magstadt DR, Meng X-J. Dendritic cell-targeted porcine reproductive and respiratory syndrome virus (PRRSV) antigens adjuvanted with polyinosinic-polycytidylic acid (poly (I: C)) induced non-protective immune responses against heterologous type 2 PRRSV challenge in pigs. Veterinary immunology and immunopathology. 2017;190:18-25.

42. Njongmeta LM, Bray J, Davies CJ, Davis WC, Howard CJ, Hope JC, et al. CD205 antigen targeting combined with dendritic cell recruitment factors and antigen-linked CD40L activation primes and expands significant antigen-specific antibody and CD4+ T cell responses following DNA vaccination of outbred animals. Vaccine. 2012;30(9):1624-35.

43. Jáuregui-Zúñiga D, Pedraza-Escalona M, Espino-Solís GP, Quintero-Hernández V, Olvera-Rodríguez A, Díaz-Salinas MA, et al. Targeting antigens to Dec-205 on dendritic cells induces a higher immune response in chickens: Hemagglutinin of avian influenza virus example. Research in veterinary science. 2017;111:55-62.

44. Mucksová J, Plachý J, Staněk O, Hejnar J, Kalina J, Benešová B, et al. Cytokine response to the RSV antigen delivered by dendritic cell-directed vaccination in congenic chicken lines. Veterinary research. 2017;48(1):1-14.

45. Jáuregui-Zúñiga D, Pedraza-Escalona M, Merino-Guzman R, Possani LD. Construction and expression of a single-chain variable fragment antibody against chicken DEC 205 for targeting the bacterial expressed hemagglutinin-neuraminidase of Newcastle disease virus. Veterinary immunology and immunopathology. 2019;212:9-14.

46. Bustamante-Córdova L, Reséndiz-Sandoval M, Hernández J. Evaluation of a Recombinant Mouse X Pig Chimeric Anti-Porcine DEC205 Antibody Fused with Structural and Nonstructural Peptides of PRRS Virus. Vaccines. 2019;7(2):43.

47. Melgoza-González EA, Reséndiz-Sandoval M, Hinojosa-Trujillo D, Hernández-Valenzuela S, García-Vega M, Mata-Haro V, et al. Antigen Targeting of Porcine Skin DEC205+ Dendritic Cells. Vaccines. 2022;10(5):684.

48. Chrun T, Lacôte S, Urien C, Richard C-A, Tenbusch M, Aubrey N, et al. A DNA vaccine encoding the gn ectodomain of Rift Valley fever virus protects mice via a humoral response decreased by dec205 targeting. Frontiers in immunology. 2019;10:860.

49. Devriendt B, Verdonck F, Summerfield A, Goddeeris BM, Cox E. Targeting of Escherichia coli F4 fimbriae to Fcγ receptors enhances the maturation of porcine dendritic cells. Veterinary immunology and immunopathology. 2010;135(3-4):188-98.

50. Subramaniam S, Cao D, Tian D, Cao QM, Overend C, Yugo DM, et al. Efficient priming of CD4 T cells by Langerin-expressing dendritic cells targeted with porcine epidemic diarrhea virus spike protein domains in pigs. Virus research. 2017;227:212-9.

51. Subramaniam S, Yugo DM, Heffron CL, Rogers AJ, Sooryanarain H, LeRoith T, et al. Vaccination of sows with a dendritic cell-targeted porcine epidemic diarrhea virus S1 protein-based candidate vaccine reduced viral shedding but exacerbated gross pathological lesions in suckling neonatal piglets. Journal of General Virology. 2018;99(2):230-9.

52. Zhang F, Zhang Z, Li X, Li J, Lv J, Ma Z, et al. Immune Responses to Orally Administered Recombinant Lactococcus lactis Expressing Multi-Epitope Proteins Targeting M Cells of Foot-and-Mouth Disease Virus. Viruses. 2021;13(10):2036.

53. Wu Y, Yan C, He Ja, Xiong W, Wu S, Liu S, et al. Reversible mannosylation as a covalent binding adjuvant enhances immune responses for porcine circovirus type 2 vaccine. ACS Omega. 2018;3(12):17341-7.

54. Huang J, Liu H, Wang M, Bai X, Cao J, Zhang Z, et al. Mannosylated gelatin nanoparticles enhanced inactivated PRRSV targeting dendritic cells and increased T cell immunity. Veterinary Immunology and Immunopathology. 2021;235:110237.

55. Renu S, Feliciano-Ruiz N, Patil V, Schrock J, Han Y, Ramesh A, et al. Immunity and protective efficacy of mannose conjugated chitosan-based influenza nanovaccine in maternal antibody positive pigs. Frontiers in immunology. 2021;12:584299.

56. Gil F, Pérez-Filgueira M, Barderas MG, Pastor-Vargas C, Alonso C, Vivanco F, et al. Targeting antigens to an invariant epitope of the MHC Class II DR molecule potentiates the immune response to subunit vaccines. Virus research. 2011;155(1):55-60.

57. Aguirreburualde MSP, Gómez MC, Ostachuk A, Wolman F, Albanesi G, Pecora A, et al. Efficacy of a BVDV subunit vaccine produced in alfalfa transgenic plants. Veterinary immunology and immunopathology. 2013;151(3-4):315-24.

58. Pecora A, Malacari DA, Aguirreburualde MSP, Bellido D, Escribano JM, Santos MJD, et al. Development of an enhanced bovine viral diarrhea virus subunit vaccine based on E2 glycoprotein fused to a single chain antibody which targets to antigen-presenting cells. Revista Argentina de Microbiología. 2015;47(1):4-8.

59. Bellido D, Baztarrica J, Rocha L, Pecora A, Acosta M, Escribano JM, et al. A novel MHC‐II targeted BVDV subunit vaccine induces a neutralizing immunological response in guinea pigs and cattle. Transboundary and Emerging Diseases. 2021;68(6):3474-81.

60. Legisa DM, Aguirreburualde MP, Gonzalez F, Marin-Lopez A, Ruiz V, Wigdorovitz A, et al. An experimental subunit vaccine based on Bluetongue virus 4 VP2 protein fused to an antigen-presenting cells single chain antibody elicits cellular and humoral immune responses in cattle, guinea pigs and IFNAR (−/−) mice. Vaccine. 2015;33(22):2614-9.

61. Borrego B, Argilaguet JM, Pérez-Martín E, Dominguez J, Pérez-Filgueira M, Escribano JM, et al. A DNA vaccine encoding foot-and-mouth disease virus B and T-cell epitopes targeted to class II swine leukocyte antigens protects pigs against viral challenge. Antiviral research. 2011;92(2):359-63.

62. Argilaguet J, Pérez-Martín E, Gallardo C, Salguero F, Borrego B, Lacasta A, et al. Enhancing DNA immunization by targeting ASFV antigens to SLA-II bearing cells. Vaccine. 2011;29(33):5379-85.

63. Grodeland G, Fredriksen AB, Løset GÅ, Vikse E, Fugger L, Bogen B. Antigen targeting to human HLA class II molecules increases efficacy of DNA vaccination. The Journal of Immunology. 2016;197(9):3575-85.

64. Mwangi W, Brown WC, Splitter GA, Davies CJ, Howard CJ, Hope JC, et al. DNA vaccine construct incorporating intercellular trafficking and intracellular targeting motifs effectively primes and induces memory B-and T-cell responses in outbred animals. Clinical and Vaccine Immunology. 2007;14(3):304-11.

65. Revilla C, Poderoso T, Martínez P, Álvarez B, López-Fuertes L, Alonso F, et al. Targeting to porcine sialoadhesin receptor receptor improves antigen presentation to T cells. Veterinary research. 2009;40(3):1-15.

66. Ooms K, Van Gorp H, Botti S, Van Gaever T, Delputte PL, Nauwynck HJ. Evaluation of viral peptide targeting to porcine sialoadhesin using a porcine reproductive and respiratory syndrome virus vaccination-challenge model. Virus research. 2013;177(2):147-55.

67. Deloizy C, Fossum E, Barnier-Quer C, Urien C, Chrun T, Duval A, et al. The anti-influenza M2e antibody response is promoted by XCR1 targeting in pig skin. Scientific reports. 2017;7(1):1-12.

68. Li K, Bao H, Wei G, Li D, Chen Y, Fu Y, et al. Molecular vaccine prepared by fusion of XCL1 to the multi-epitope protein of foot-and-mouth disease virus enhances the specific humoural immune response in cattle. Applied microbiology and biotechnology. 2017;101(21):7889-900.

69. Bernelin-Cottet C, Urien C, Fretaud M, Langevin C, Trus I, Jouneau L, et al. A DNA prime immuno-potentiates a modified live vaccine against the porcine reproductive and respiratory syndrome virus but does not improve heterologous protection. Viruses. 2019;11(6):576.
